# Man-Made Synthetic Receptors for Capture and Analysis of Ochratoxin A

**DOI:** 10.3390/toxins7104083

**Published:** 2015-10-10

**Authors:** Claudio Baggiani, Cristina Giovannoli, Laura Anfossi

**Affiliations:** 1 Laboratory of Bioanalytical Chemistry, Department of Chemistry, University of Torino, Torino 10125, Italy; E-Mails: cristina.giovannoli@unito.it (C.G.); laura.anfossi@unito.it (L.A.); 2 Nanostructured Interfaces and Surfaces Interdepartmental Centre, University of Torino, Torino 10125, Italy

**Keywords:** mycotoxin, Ochratoxin A, molecularly imprinted polymer, aptamer, oligosorbent, peptide library, binding peptide, solid phase extraction

## Abstract

Contemporary analytical methods have the sensitivity required for Ochratoxin A detection and quantification, but direct application of these methods on real samples can be rarely performed because of matrix complexity. Thus, efficient sample pre-treatment methods are needed. Recent years have seen the increasing use of artificial recognition systems as a viable alternative to natural receptors, because these materials seem to be particularly suitable for applications where selectivity for Ochratoxin A is essential. In this review, molecularly imprinted polymers, aptamers and tailor-made peptides for Ochratoxin A capture and analysis with particular attention to solid phase extraction applications will be discussed.

## 1. Introduction

**Figure 1:**
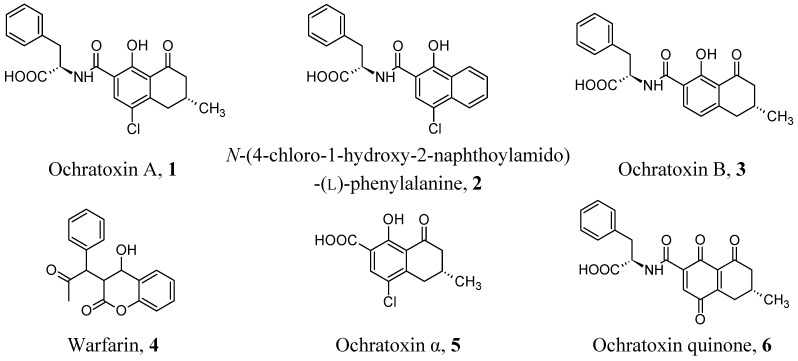
Molecular structures of OTA and related compounds cited in this review.

Nowadays, it is largely accepted in academic circles and public health bodies that food and feed contamination from Ochratoxin A (OTA, **1**, Figure 1) is a severe public health problem [1,2]. In fact, OTA can deeply affect health not only after a single massive exposure but, more often, after continuous exposure to low doses, and that such exposure can be related to several chronic diseases, including some types of cancer and serious renal and immunological dysfunctions [3,4,5,6]. Thus, good analytical protocols based on efficient analytical processes, sensitive, selective, fast, inexpensive and suitable for sample mass screenings, are required by legislation, health authorities and companies operating in the food market [7,8,9].

At present, commercially available rapid assays for OTA based on the use of immunoanalytical techniques are widely diffused, as these analytical techniques assure the feasibility of fast sample mass screenings in an affordable fashion [10,11]. However, a sample which is positive for OTA contamination should be validated by using more sophisticated analytical methods [12,13,14]. These methods are usually based on instrumental separative techniques coupled with mass spectrometric detectors of varying complexity. They have the sensitivity required for contamination detection and quantification, but direct application of these techniques on food and feed samples can be rarely performed. In fact, OTA is usually present in food at very low concentration (ng-µg/kg) levels, dispersed in highly complex and morphologically structured matrices, with an elevated degree of point-to-point and sample-to-sample variability. Thus, such a type of matrix introduces severe disturbances in the analytical separation step, and quantitative analysis can be performed only after extensive clean-up and preconcentration steps.

Current sample pre-treatment methods, mostly based on the solid phase extraction technique, are very fast and economical but not selective, while methods based on immunoaffinity extraction and utilising on-line columns or off-line cartridges are very selective but expensive and usually not suitable for harsh environments and columns recycling [15,16]. Thus, economical, rapid and selective clean-up methods based on “intelligent” materials are needed. At present, as reported in Table 1, tailor-made, artificial systems as molecularly imprinted polymers, aptamers and binding peptides obtained by combinatorial synthesis are good candidates to circumvent the drawbacks typical of more traditional solid phase extraction techniques. These materials seem to be particularly suitable for extractive applications where OTA-selectivity in the presence of very complex samples represents the main problem. The main goal of this review is to examine the application of such different tailor-made materials in the analysis of food contamination by OTA with particular attention to solid phase extraction applications.

**Table 1:** A comparison between immunoaffinity extraction and techniques based on artificial receptors.

Issues	Immunoaffinity Chromatography	Molecularly Imprinted Polymers	Aptamers	Binding Peptides
binding structures	anti-OTA antibodies grafted onto chromatography-type solid supports	cross-linked synthetic polymers prepared in presence of OTA or OTA-mimic molecules	oligonucleotides selected through a sequential affinity purification/PCR amplification process	linear peptides selected through a sequential screening of a of a spatially addressable peptide combinatorial library
binding affinity	high	medium	medium	low
binding site density	low	high	low	low
binding kinetics	slow dissociation	slow dissociation	fast dissociation	fast dissociation
binding selectivity	high	high	high	high
reproducibility	limited	very high	very high	very high
non-specific binding	negligible	significant in water	negligible	negligible
resistance to extreme pH	no	yes	no	no
resistance to organic solvents	limited	yes	yes	yes
resistance to denaturing agents	no	yes	yes	yes
resistance to microorganisms	no	yes	no	no
needs of a solid support	yes	no	yes	yes
reuse	difficult, mainly monouse	yes	yes	yes
costs	low to medium	low	low	low
commercial availability as read-to-use	yes	yes	no	no
literature	large	growing	growing	limited

## 2. Molecularly Imprinted Polymers

**Figure 2:**
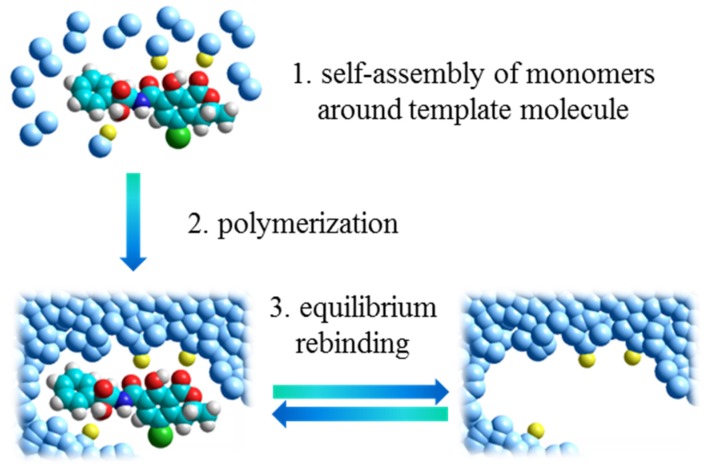
The molecular imprinting process.

Molecularly imprinted polymers (MIPs) are synthetic materials characterized by the presence of binding sites able to selectively recognize a target molecule [17,18,19]. As illustrated in Figure 2, these materials are synthetized by polymerization around a template molecule of cross-linkers and functional monomers able to interact with the functional groups of the template through non-covalent interactions. Once polymerization has taken place, a highly cross-linked three-dimensional network polymer is formed and binding sites with shape, size and functionalities complementary to the template are established in the bulk of the polymer. These artificial binding sites have the same features as antibody binding sites, showing binding reversibility, enhanced selectivity and high affinity constant. Such features make MIPs very popular as binding materials in the so-called MISPE (Molecularly Imprinted Solid Phase Extraction) approach. This technique is similar to the traditional immunoextraction based on natural antibodies. A small amount of imprinted polymer (typically 25–500 mg) is packed in an open column (for off-line applications) or in a closed HPLC cartridge (for on-line applications). Then, the usual steps of column conditioning, sample loading, column washing and analyte elution are performed [20,21].

In the last fifteen years, a growing number of papers dealing with MISPE has been dedicated to the clean-up and preconcentration of mycotoxins in complex matrices [22], and OTA is the first mycotoxin for which a successful molecular imprinting was reported in literature [23,24].

### 2.1. OTA Imprinting by Template Mimics

The main critical point associated with the development of MISPE protocols for OTA is represented by the residual template not being completely removed from the polymeric matrix and slowly leaking during loading, washing and elution operations. Such a template loss (“bleeding effect”) can be detected at trace levels during the elution step, and it represents a significant source of interferences and systematic errors in trace analysis of OTA. The most successful strategy to avoid the bleeding effect consists in the use of a structural analogue of the OTA molecule in the so-called “template mimic” approach, where the choice of the mimic template should be made in such a way as to obtain imprinted binding sites provided with good selectivity towards OTA but, at the same time, this structural analogue should be different from OTA in such a way that the analytical separation performed after the extraction step discriminates clearly between OTA and the residual template molecules released by the imprinted material.

To prepare a mimic of OTA, the same synthetic strategy has been independently reported by Baggiani *et al.* [23] and Jodlbauer *et al.* [24]. A good mimic template was rationally designed to preserve the general structure of the target analyte, including the chirality of the amino acidic sub-structure and the planarity of the benzopyranic sub-structure. At the same time, the α-unsaturated lactone moiety characterizing many carcinogenic mycotoxins was eliminated, while the distinct points of potential interaction with functional monomers were maintained: the α-carboxyl of L-phenylalanine, the amide bridge, and the phenolic hydroxyl. The resulting mimic template, *N*-(4-chloro-1-hydroxy -2-naphthoylamido)-(l)-phenylalanine (**2**), showed almost complete overlapping of the two molecules, with a high degree of similarity not only as structures, but also as solvent accessible surfaces, electrostatic potential surfaces and lipophilic/hydrophilic surfaces. Different polymerization mixtures were considered to prepare the polymers. In fact, while Baggiani *et al.* obtained an imprinted polymer using a methacrylic acid/ethylenedimethacrylate mixture, Jodlbauer *et al.* used a more exotic mixture with quinuclidine methacrylamide and tert-butylmethacrylamide as functional monomer and ethylene dimethacrylate as cross-linker. Anyway, both the approaches proved valid, with the presence of specific molecular recognition effects due to hydrogen bond interactions and steric factors and good recognition of OTA compared to several analogs in polar (methanol, acetonitrile) and hydrophobic (chloroform) solvents.

The effect of different template mimics derived by **2** on the molecular recognition properties of the resulting imprinted polymers towards OTA has been studied further [25]. The experimental results show that changes to the amino acidic sub-structure or the presence/absence of a chlorine atom in position 4 on the naphthalene ring system does not affect the molecular recognition of OTA by the resulting imprinted polymer. On the contrary, the presence of the bulky naphthalene ring system in the mimic template seems to be necessary to preserve the molecular recognition of OTA.

The quinuclidine methacrylamide polymer reported by Jodlbauer *et al.* was used in a subsequent work to extract OTA from red wine before quantification by HPLC-fluorescence detection [26]. The approach involved a two-stage sample clean-up protocol on coupled reversed-phase (C18-silica) and MISPE cartridges, where the use of the reversed-phase cartridge was crucial for the removal of the interfering acidic matrix compounds. The method provided recovery >90% and a relative standard deviation <10%, with detection and quantification limits of 10 and 33 ng/L in spiked and commercial red wines. However, the authors raised doubt on the validity of the MISPE protocol, as identical performances were observed when the imprinted polymer was replaced by the corresponding non-imprinted material.

On the contrary, the validity of the template mimic-based MISPE approach to OTA analysis in wine by HPLC-fluorescence detection has been recently confirmed by Giovannoli *et al.* [27]. Under optimized conditions, the authors extracted and determined OTA from 17 red wines from different geographical regions of Italy, with detection and quantification limits, respectively, of 75 and 225 ng/L, and recoveries ranging from 88% to 102%. Wine samples determined by immunoaffinity extraction showed the MISPE method to be comparable, proving the potential of such approach to substitute for the current immunoaffinity method.

The template mimic 2 has been also used for the development of an automatic MISPE system coupled with a fluorescence detector for the sensitive determination of OTA in wheat [28]. The on-line extraction demonstrated the efficient cleanup of the matrix by the imprinted column and the detection of OTA in wheat samples in the range 3–18 µg/L, with a limit of detection of 1.2 µg/L and recoveries of 93% ± 9%.

### 2.2. Direct OTA Imprinting

The direct use of OTA as template has been described by Turner *et al.* [29]. In this case, an *in silico*-designed prepolymerization mixture was used to optimize the non-covalent interactions between the template molecule and the functional monomers. From these simulations, a mixture of methacrylic acid and acrylamide was selected as functional monomers, while ethylene dimethacrylate was used as cross-linker. The use of N,Nʹ-dimethylformamide as a porogenic solvent, uncommon in molecular imprinting, generated a pH-responsive imprinted polymer with excellent affinity and specificity for OTA in acidic aqueous solutions, while more basic conditions caused a loss of recognition properties. Unfortunately, no applications in food clean-up were reported by the authors for this polymer.

An uncommon functional monomer (*N*-phenylacrylamide) was used by Zhou *et al.* to prepare an OTA-imprinted polymer suitable for on-line MISPE and fluorescence detection of OTA in wheat extracts [30]. The authors showed that pulsed elution by using 20-µL spikes of methanol/triethylamine (99:1 *v*/*v*) was good for the quantitative desorption of OTA from the MISPE cartridge, affording a detection limit of 5 µg/L and a mean recovery from wheat extracts of 103% ± 3%.

### 2.3. OTA Imprinting in Polypyrrole Layers

In a completely different approach with respect to traditional imprinted polymers, thin layers of OTA-imprinted electropolymerized polypyrrole were used to set-up on-line MISPE miniaturized devices for the detection and quantification of OTA in wine. The imprinted polypyrrole layers were supported on stainless-steel frits, directly grafted onto the porous steel surface [31] or previously adsorbed onto single-wall carbon nanotubes to enhance the binding capacity of the imprinted layer [32,33]. When relatively large amount of wine samples, up to 3 mL, were loaded onto the extraction devices, selective recoveries up to 40% was obtained when frits were eluted with 20-µL spikes of methanol/triethylamine (99:1 *v*/*v*), with detection limits of 50 ng/L and 12 ng/L for a polypyrrole-imprinted layer supported by steel and single-wall carbon nanotubes respectively.

The use of carbon nanotube-supported imprinted polypyrrole layers as recognition element has been used to prepare a micro-solid phase preconcentration device packed inside a 22-gauge syringe needle by Wei *et al.* [34]. Using a sample volume of 0.5 mL of red wine for preconcentration, it was possible to determine OTA by HPLC with fluorescence detection down to a detection limit of 40 ng/L, with a quantification limit of 100 ng/L with very short times of extraction and instrumental analysis.

### 2.4. Commercial OTA-Imprinted Polymers

Besides experimental polymers prepared by research groups, recently, several papers have been published where commercially-available OTA-imprinted polymers has been used to successfully set-up and optimize MISPE protocols for OTA in several foods. Even if the commercial source does not declare the nature of the template used to prepare such MIPs, on the grounds of the published results, it is reasonable to assume that it can be the mimic template **2** or a strictly correlated structure. Commercial MISPE cartridges have been validated for the extraction and analysis of OTA in cereals by HPLC (LOD: 2.5 µg/Kg) [35], coffee, grape juice and urine by micro-solid phase extraction and HPLC (LOD: 60, 20 and 20 ng/Kg, respectively) [36], ginger by HPLC-MS/MS (LOD: 90 ng/L) [37], beer, red wine, and grape juice by HPLC (LOD: 25 ng/L) [38], urine by micro-solid phase extraction and capillary electrophoresis (LOD: 11 µg/L) [39], and by HPLC (LOD: 0.2 µg/L) [40]. Moreover, commercial MISPE cartridges have been compared with commercial immunoaffinity columns and Oasis^®^ HLB cartridges for the extraction of OTA from wine, beer, roasted coffee and chili, showing that MISPE cartridges were comparable to the other extraction cartridges in the analytical performances (recovery, reproducibility, limit of detection and limit of quantification) for all the matrices considered except for coffee, where proved superior [41].

## 3. Aptamers

Aptamers are artificial single-strand DNA (ssDNA) or RNA (ssRNA) oligonucleotides whose sequence, typically composed by 20–100 base pairs, show molecular recognition properties towards target molecules, characterized by high affinity and marked selectivity. Such binding properties are originated by the folding of the sequence in complex three-dimensional structures, characterized by the presence of structural motifs as stems, loops, bulges, hairpins, pseudoknots, triplexes. The unique base sequence of each aptamer assures that structural motifs are peculiar and complementary to the aptamer’s molecular target [42].

**Figure 3:**
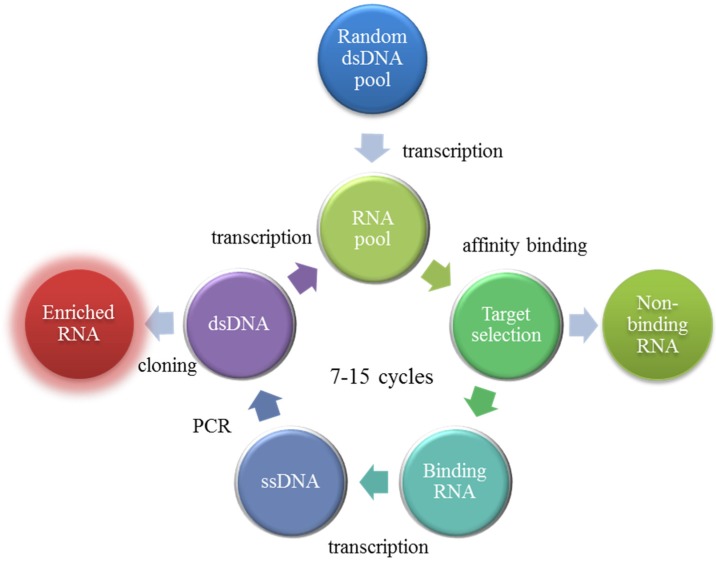
The SELEX process.

Aptamers can be obtained through an *in vitro* selection and amplification procedure called SELEX (Systematic Evolution of Ligands by Exponential Enrichment). It consists of an iterative process (typically, 7 to 15 repeated steps) alternating between ligand selection and sequence amplification. As reported in Figure 3, the process starts from the synthesis of a combinatorial library of single strand oligonucleotides where each sequence is structured by two primer-binding regions at each end and a central region of 20 to 80 nucleotides in random sequence. During the selection, the target molecule is incubated with the oligonucleotide library and the best binding sequences are separated by affinity chromatography and amplified by a polymerase chain reaction. By process iteration, as direct effect of the increasingly stringent conditions of binding/release required for the oligonucleotides in the chromatographic step, the molecular complexity of the library is progressively reduced and it is enriched of high affinity sequences to isolate the best binding oligonucleotide, which can be sequenced and synthetized in bulk quantities [43,44].

Because of their peculiar binding properties, aptamers appear to be an interesting alternative to antibodies in several analytical applications [45], including not only enzyme-linked oligonucleotide assays (a variant of ELISA with aptamers instead of antibodies) [46] or biosensors (aptasensors) [47] but also oligonucleotides-based sorbents (oligosorbents) in chromatography and capillary electrophoresis [48,49].

### OTA-Binding Aptamers as Oligosorbents

Even if the use of aptamers as oligosorbents is very recent, their application to sample containing OTA appears to be a promising analytical application of such technology. The first reported aptamer-based solid phase extraction of OTA was reported by Cruz-Aguado and Penner in 2008 [50]. The authors described the identification by SELEX procedure of several ssDNAs, characterized by dissociation affinity constants towards OTA measured between 19.5 and 0.36 µM (Table 2). The selectivity of the best binding sequence, named aptamer 1.12, was studied by competition of OTA with Ochratoxin B (the dealogenated mycotoxin, **3**), *N*-acetylphenylalanine (related to the aminoacidic sub-structure of the mycotoxin) and warfarin, **4** (similar to Ochratoxin α, **5**, the isocoumarinic sub-structure of the mycotoxin), and it was found that aptamer 1.12 did not bound *N*-acetylphenylalanine nor warfarine, while it bound Ochratoxin B 100-fold less than OTA. The subsequent removal of the forward primer in the sequence of 1.12, a section of the 5′-nonconsensus sequence and three bases from the 3′-end, resulted in the aptamers 1.12.2 and 1.12.8, characterized by a significant increase in binding affinity from 0.36 to 0.2 µM. The solid phase extraction cartridge grafted with the aptamer 1.12.2 was used to extract OTA from food samples. The method proved to be successful when used for certified naturally contaminated wheat samples containing OTA at levels from 1.8 to 61.9 µg/Kg, with recovery between 81% and 136% and reproducibility between 0.4% and 9.0%.

**Table 2:** The Ochratoxin A (OTA)-binding aptamer sequences obtained by SELEX (systematic evolution of ligands by exponential enrichment) protocol reported in [50]. For clarity, the sequence of the primers was omitted and bases conserved across all sequences are highlighted in red.

Aptamer	Sequence	K_d_ (µM)
**1.12**	GCATCTGATCGGGTGTGGGTGGCGTAAAGG	0.36
**1.12.2**	GATCGGGTGTGGGTGGCGTAAAGGGAGCATCGGACA	0.2
**1.12.5**	GATCGGGTGTGGGTGGCGTAAAGGGAGCATCGGACAACG	0.8
**1.12.8**	GATCGGGTGTGGGTGGCGTAAAGGGAGCATCGG	0.2
**1.12.9**	GATCGGGTGTGGGTGGCGTAAAGGGAGCAT	1.6
**1.12.11**	GATCGGGTGTGGGTGGCGTAAAGGGAGCATCG	0.4
**1.12.12**	GATCGGGTGTGGGTGGCGTAAAGGGAGCATC	0.5
**1.13**	GGGGTGAAACGGGTCCCG	6.7
**1.14**	GCAGTCCTAGATCGGGTGTGGCTGGCTTGG	0.99
**1.4**	GCACGATGGGGAAAGGGTCCCCCTGGGTTG	19.3
**2.2**	ACTGTCCGTCGGGTTTAGGGTGGCATTGG	1.6
**2.3**	TCAGTCCCGATCA GGTGTGGGTGGC ATTGG	1.7
**2.4**	CCAAATCGGACGGGGCCTGTTTTAATGGGG	19.5
**2.6**	CGTACGGTGGGAACGGTTCCTCTTAGGGT	7.1
**2.9**	CAGGTGGCAGATCGGGTGTGGGTGGCCTGG	0.96
**2.10**	ACATGCGACTGAGGCTCGGTTTATTGAGGG	4.3
**2.11**	CCTGACGATCGGGTGTGGGTTGGCTTGAGG	2.5
**2.12**	CCTTGTAGATCGGGTGTGGTTTGGCGTAGG	0.97
**2.13**	GCAGTACGATCGGGGGTGGGTGGATGTAGG	1.9

The effect of the immobilization strategy used to graft the aptamer 1.12.2 onto the solid support was studied by Chapuis-Hugon *et al.* by considering a noncovalent binding by using streptavidin-activated agarose with a 5′-biotinylated aptamer, and a covalent binding by using cyanogen bromide-activated Sepharose with a 5′-amino-modified aptamer [51]. The resulting oligosorbents were evaluated in terms of retention, selectivity, and binding capacity, finding no significant differences between both of the approaches. However, when the solid phase extraction protocol was applied to wine samples, the covalent grafting was found more robust. In a later work [52], the oligosorbent prepared by covalent grafting was modified by changing the side of the oligonucleotide chain bound to the solid support, the length of the spacer arm and the amount of the aptamer grafted onto the solid support. Good extraction performances were obtained on all the solid supports, without any cross-reactivity towards OTA metabolites such Ochratoxin B and Ochratoxin quinone (**6**). The oligosorbent were applied to the clean-up of OTA from wheat sample extracts with quantitative extraction recoveries and complete removal of matrix components.

Solid phase extraction of OTA by using aptamer 1.12.2 grafted on solid supports in off-line modality and HPLC analysis with fluorescence detection has been reported for several food samples. De Girolamo *et al.* compared HPLC analyses of 33 naturally contaminated durum wheat samples cleaned-up on both oligosorbent and immunoaffinity columns [53], showing a good correlation coefficient (*r* = 0.990), with average recoveries at levels of 0.5–50 µg/Kg ranging from 74% to 88%, and with limits of detection and of quantification of 23 and 77 ng/Kg, respectively. Rhouati *et al.* measured OTA in beers by direct extraction with a limit of detection of 0.2 ng/ml and an average recovery of 96% [54]. Yang *et al.* measured OTA in ginger powder after extraction and clean-up with average recoveries at levels of 5, 15, and 45 µg/Kg ranging from 85.4% to 96.8%, confirming obtained results by UHPLC-MS/MS [55].

Magnetic solid-phase extraction was used by Wu *et al.* to extract OTA from wheat flour, coffee and cereals samples [56]. The authors used an oligosorbent prepared by covalent grafting the aptamer 1.12.2 onto magnetic ferrite nanospheres. The efficacy of the approach was successfully evaluated through comparison with conventional solid-phase extraction on commercial C18 cartridge of food samples containing OTA in the range of 2.5–50 µg/Kg, yielding recoveries from 67% to 90%, respectively. The validated extraction method was successfully applied to 52 unfortified food samples, finding positive contaminations at levels of 2.7–5.8 µg/Kg for three of them.

Yang *et al.* developed a UHPLC-MS/MS method for the determination of OTA in traditional Chinese drugs, by using vortex-assisted solid-liquid microextraction and aptamer-affinity column to extract samples before the analysis [57]. Both the clean-up approaches resulted in low limit of detection (0.5–0.8 µg/Kg), and satisfactory recovery (83.5%–94.4%). Moreover, the oligosorbent showed an excellent selectivity owing to its specific identification of OTA in various kinds of selected drugs and eliminating the matrix effects for the MS/MS analysis.

A miniaturized oligosorbent for OTA was prepared via the *in situ* sol-gel synthesis of a hybrid organic-inorganic monolith in 100 µm i.d. capillary columns using organosilanes as precursors, followed by covalent grafting of the aptamer 1.12.2 [58]. The oligosorbent was coupled on-line to a nanoLC with lased induced fluorescence detector, and selective extraction of OTA was demonstrated with an average extraction recovery above 80%. The capillary device was applied to the extraction of OTA from beer samples, appearing effective to isolate OTA from the matrix.

## 4. Combinatorial Peptides

Synthetic molecular recognition systems can be obtained by combinatorial chemistry applied to peptide synthesis, which allows to prepare very large libraries of peptides with well-characterized binding properties. The roots of combinatorial organic synthesis stem from the development of solid-phase peptide synthesis (SPPS) by Merrifield [59]. Solid-phase peptide synthesis consists of sequentially coupling amino acid monomers onto a growing peptide chain, which is immobilized on a solid support. The primary advantages of solid-phase synthesis are related with the possibility to add a large excess of activated amino acids to drive peptide coupling reactions to completion, and purification protocols greatly facilitated by covalent attachment of the intermediate products to the insoluble support. Thus, nowadays, SPPS provides an efficient method to prepare large peptides libraries by combinatorial approaches. Such libraries are characterized by defined sequences and elevated molecular diversity, assuring a great potential to recognize and capture molecular recognition targets.

### OTA-Binding Combinatorial Peptides

**Figure 4:**
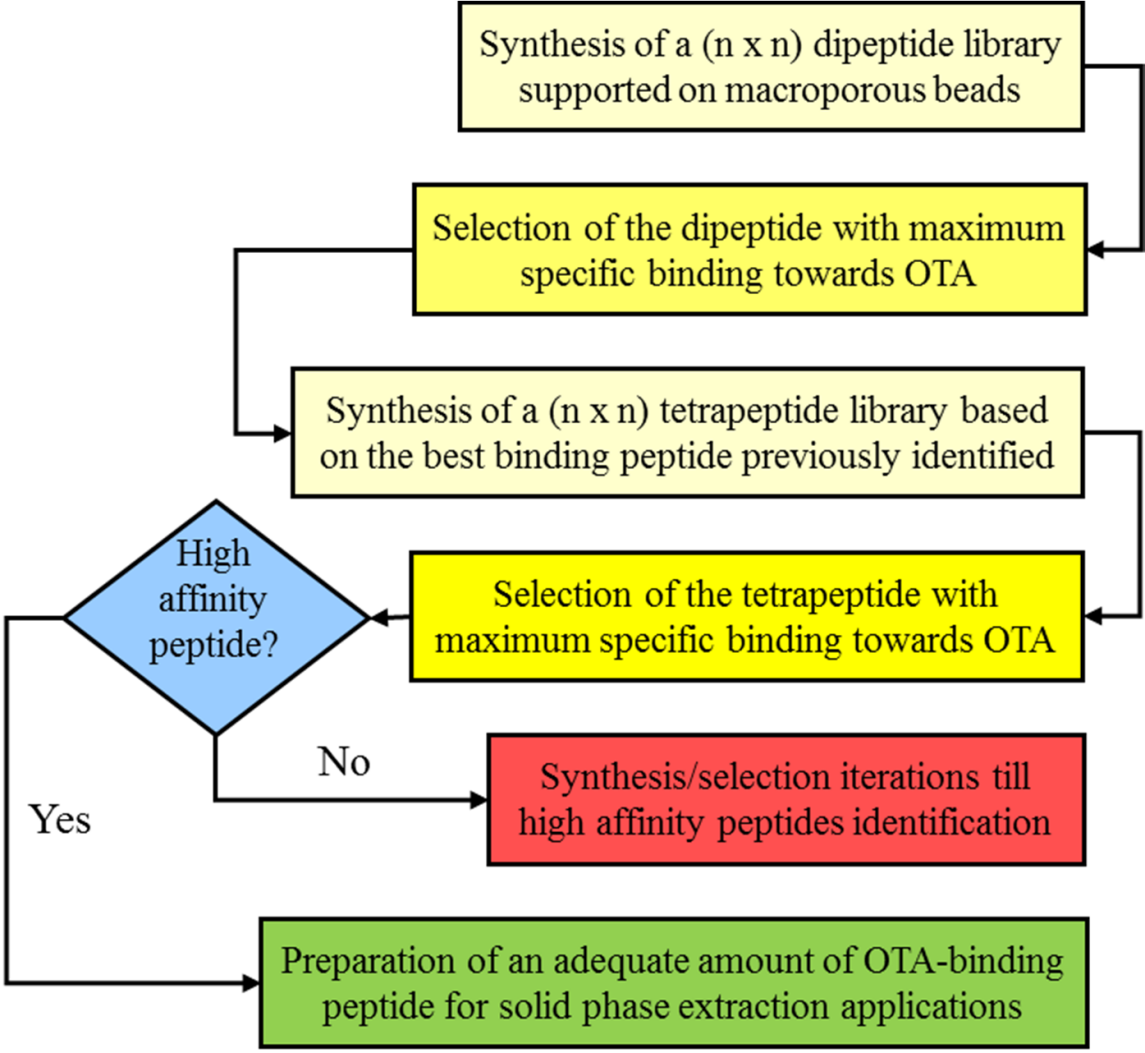
Scheme of the sequential development of a spatially addressable parallel OTA-binding peptide library.

Notwithstanding the huge popularity of the SPPS approach, the present literature is principally related to the search for sequences with molecular recognition properties towards biomacromolecules with biotechnological or medical implications [60,61], while libraries with recognition towards small ligands are rare and mainly directed towards the use of peptides for separative processes [62]. Thus, it is not surprising that it is very difficult to find literature on peptides with molecular recognition towards mycotoxins. On this premise, our laboratory has developed a target-focused strategy for the fast preparation of synthetic peptide libraries [63,64,65,66]. This strategy is based on the sequential development of a spatially addressable parallel library where, after each peptide elongation step, the binding properties towards the target molecule are measured, and only the peptide with the best binding behavior and selectivity is retained and selected as a scaffold for the successive elongation step, while the other sequences are discarded (see Figure 4).

Peptides with molecular recognition properties towards OTA were identified in a 12 × 12 library containing the amino acids Ala, Arg, Asn, Phe, Gly, His, Leu, Lys, Pro, Ser, Trp and Val [65]. The first amino acid library composed of 144 different dipeptides showed as best binding sequence the dipeptide Ser-Asn, with an dissociation affinity constant for the target of 0.12 mM. A tetrapeptide library developed by using the sequence Ser-Asn as scaffold showed a general increasing of the affinity for OTA, with the sequence Ser-Asn-Leu-His with an dissociation affinity constant of 77 µM. The binding performances were developed further by preparing an hexapeptide library where the peptide Ser-Asn-Leu-His-Pro-Lys showed a dissociation affinity constant of 29 µM. OTA binding by the hexapeptide was found to be pH-regulated with the maximum binding for mildly acidic buffers (pH 3–4), while more acidic buffer (pH 2.2) reduced the binding and weak, neutral or alkaline buffers (pH 5–9) showed a marked binding inhibition.

Beads grafted with the OTA-binding peptide were used to set up a solid phase extraction method for OTA in wines at concentration levels down to 0.10 µg/L. Several different red, white and rosé wine samples fortified with the mycotoxin showed recovery of 95% and 98% at 2.0 and 4.0 µg/L, respectively, without any effect on the extraction efficiency of the matrix. The efficacy of this approach was successfully tested by comparison with an immunoaffinity extraction performed on commercial immunosorbents.

## 5. Conclusions

As shown in the previous sections, several different approaches can be successfully used to prepare artificial receptors for detection, clean up and preconcentration of OTA in complex samples. The ever growing literature shows that the development of such receptors can be considered of relevant scientific and practical interest. High selectivity, long-term stability and low costs of preparations make all them good competitors towards traditional antibody-based solid phase extraction materials. While MIP-based supports are already commercially available, oligosorbents and peptides are not, but considering their binding properties and the full compatibility with aqueous samples, it is reasonable to expect that, in the near future, they could represent viable alternatives to both MIPs and natural antibodies.

## References

[1] Duarte S.C., Lino C.M., Pena A. (2010). Mycotoxin food and feed regulation and the specific case of Ochratoxin A: A review of the worldwide status. Food Additiv. Contam. A.

[2] El Khoury A., Atoui A. (2010). Ochratoxin A: General overview and actual molecular status. Toxins.

[3] Reddy L., Bhoola K. (2010). Ochratoxins-food contaminants: Impact on human health. Toxins.

[4] Klaric M.S., Rasic D., Peraica M. (2013). Deleterious effects of mycotoxin combinations involving Ochratoxin A. Toxins.

[5] Sorrenti V., Di Giacomo C., Acquaviva R., Barbagallo I., Bognanno M., Galvano F. (2013). Toxicity of Ochratoxin A and its modulation by antioxidants: A review. Toxins.

[6] Bezeria da Rocha M.E., Oliveira Freire F.C., Feitosa Maia F.B., Florindo Guedes M.I., Rondina D. (2014). Mycotoxins and their effects on human and animal health. Food Contr..

[7] European Commission (2003). Commission Regulation 100/03. Off. J. Eur. Commun..

[8] European Commission (2004). Commission Regulation 683/04. Off. J. Eur. Commun..

[9] European Commission (2006). Commission Regulation 1881/06. Off. J. Eur. Commun..

[10] Meulenberg E.P. (2012). Immunochemical methods for Ochratoxin A detection: A review. Toxins.

[11] Anfossi L., Baggiani C., Giovannoli C., D’Arco G., Giraudi G. (2013). Lateral-flow immunoassays for mycotoxins and phycotoxins: A review. Anal. Bioanal. Chem..

[12] Turner N.W., Subrahmanyam S., Piletsky S.A. (2009). Analytical methods for determination of mycotoxins: A review. Anal. Chim. Acta.

[13] Berthiller F., Burdaspal P.A., Crews C., Iha M.H., Krska R., Lattanzio V.M.T., MacDonald S., Malone R.J., Maragos C., Solfrizzo M. (2014). Developments in mycotoxin analysis: An update for 2012–2013. World Mycotox. J..

[14] Berthiller F., Brera C., Crews C., Iha M.H., Krska R., Lattanzio V.M.T., MacDonald S., Malone R.J., Maragos C., Solfrizzo M. (2015). Developments in mycotoxin analysis: An update for 2013–2014. World Mycotox. J..

[15] Pichon V., Delaunay-Bertoncini M., Hennion M.C., Pawliszyn J. (2002). Immunosorbents in sample preparation. Sampling and Sample Preparation for Field and Laboratory—Comprehensive Analytical Chemistry Ser.

[16] Senyuva H.Z., Gilbert J. (2010). Immunoaffinity column clean-up techniques in food analysis: A review. J. Chromatogr. B.

[17] Sellergren B. (2001). Molecularly Imprinted Polymers: Man-Made Mimics of Antibodies and Their Applications in Analytical Chemistry.

[18] Yan M., Ramström O. (2004). Molecularly Imprinted Materials.

[19] Haupt K. (2011). Molecular Imprinting.

[20] Beltran A., Borrull F., Cormack P.A.G., Marcè R.M. (2010). Molecularly-imprinted polymers: Useful sorbents for selective extractions. Trends Anal. Chem..

[21] Martin-Esteban A. (2013). Molecularly-imprinted polymers as a versatile, highly selective tool in sample preparation. Trends Anal. Chem..

[22] Baggiani C., Anfossi L., Giovannoli C. (2008). Molecular imprinted polymers as synthetic receptors for the analysis of myco- and phyco-toxins. Analyst.

[23] Baggiani C., Giraudi G., Vanni A. (2002). A molecular imprinted polymer with recognition properties towards the carcinogenic mycotoxin Ochratoxin A. Bioseparation.

[24] Jodlbauer J., Maier M.M., Lindner W. (2002). Towards Ochratoxin A selective molecularly imprinted polymers for solid-phase extraction. J. Chromatogr. A.

[25] Baggiani C., Biagioli F., Anfossi L., Giovannoli C., Passini C., Giraudi G. (2013). Effect of the mimic structure on the molecular recognition properties of molecularly imprinted polymers for Ochratoxin A prepared by a fragmental approach. React. Funct. Polym..

[26] Maier N.M., Buttinger G., Welhartizki S., Gavioli E., Lindner W. (2004). Molecularly imprinted polymer-assisted sample clean-up of Ochratoxin A from red wine: Merits and limitations. J. Chromatogr. B.

[27] Giovannoli C., Passini C., Di Nardo F., Anfossi L., Baggiani C. (2014). Determination of Ochratoxin A in italian red wines by molecularly imprinted solid phase extraction and HPLC analysis. J. Agric. Food Chem..

[28] Vidal J.C., Duato P., Bonel L., Castillo J.R. (2012). Molecularly imprinted on-line solid-phase extraction coupled with fluorescence detection for the determination of Ochratoxin A in wheat samples. Anal. Lett..

[29] Turner N.W., Piletska E.V., Karim K., Whitcombe M., Malecha M., Magan N., Baggiani C., Piletsky S.A. (2004). Effect of the solvent on recognition properties of molecularly imprinted polymer specific for Ochratoxin A. Biosens. Bioelectron..

[30] Zhou S.N., Lai E.P.C., Miller J.D. (2004). Analysis of wheat extracts for Ochratoxin A by molecularly imprinted solid-phase extraction and pulsed elution. Anal. Bioanal. Chem..

[31] Yu J.C.C., Krushkova S., Lai E.P.C., Dabek-Zlotorzynska E. (2005). Molecularly-imprinted polypyrrole-modified stainless steel frits for selective solid phase preconcentration of Ochratoxin A. Anal. Bioanal. Chem..

[32] Yu J.C.C., Lai E.P.C. (2006). Molecularly imprinted polypyrrole modified carbon nanotubes on stainless steel frit for selective micro solid phase pre-concentration of Ochratoxin A. React. Funct. Polym..

[33] Yu J.C.C., Lai E.P.C. (2007). Determination of Ochratoxin A in red wines by multiple pulsed elutions from molecularly imprinted polypyrrole. Food Chem..

[34] Wei Y., Qiu L.H., Yu J.C.C., Lai E.P.C. (2007). Molecularly imprinted solid phase extraction in a syringe needle packed with polypyrrole-encapsulated carbon nanotubes for determination of Ochratoxin a in red wine. Food Sci. Technol. Int..

[35] Ali W.H., Derrien D., Alix F., Perollier C., Lepine O., Bayoudh S., Chapuis-Hugon F., Pichon V. (2010). Solid-phase extraction using molecularly imprinted polymers for selective extraction of a mycotoxin in cereals. J. Chromatogr. A.

[36] Lee T.P., Saad B., Khayoon W.S., Salleh B. (2012). Molecularly imprinted polymer as sorbent in micro-solid phase extraction of Ochratoxin A in coffee, grape juice and urine. Talanta.

[37] Cao J.L., Zhou S.J., Kong W.J., Yang M.H., Wan L., Yang S.H. (2013). Molecularly imprinted polymer-based solid phase clean-up for analysis of Ochratoxin A in ginger and LC-MS/MS confirmation. Food Control.

[38] Cao J.L., Kong W.J., Zhou S.J., Yin L.H., Wan L., Yang M.H. (2013). Molecularly imprinted polymer-based solid phase clean-up for analysis of Ochratoxin A in beer, red wine, and grape juice. J. Sep. Sci..

[39] Lee T.P., Saad B., Salleh B., Mat I. (2013). Micro-solid phase extraction of Ochratoxin A, and its determination in urine using capillary electrophoresis. Microchim. Acta.

[40] Xie L., Sheng P., Kong W., Zhao X., Ouyang Z., Yang M. (2015). Solid-phase extraction using molecularly imprinted polymer for determination of Ochratoxin A in human urine. World Mycotox. J..

[41] Prelle A., Spadaro D., Denca A., Garibaldi A., Gullino M.L. (2013). Comparison of clean-up methods for Ochratoxin A on wine, beer, roasted coffee and chili commercialized in Italy. Toxins.

[42] Hermann T., Patel D.J. (2000). Adaptive recognition by nucleic acid aptamers. Science.

[43] Ellington A.D., Szostak J.W. (1990). *In vitro* selection of RNA molecules that bind specific ligands. Nature.

[44] Stoltenburg R., Reinemann C., Strehlitz B. (2007). Selex—A (r)evolutionary method to generate high-affinity nucleic acid ligands. Biomol. Eng..

[45] Mascini M., Palchetti I., Tombelli S. (2012). Nucleic acid and peptide aptamers: Fundamentals and bioanalytical aspects. Angew. Chem. Int. Ed..

[46] Tombelli S., Minunni M., Mascini M. (2007). Aptamers-based assays for diagnostics, environmental and food analysis. Biomol. Eng..

[47] Song S., Wang L., Li J., Fan C., Zhao J. (2008). Aptamer-based biosensors. Trends Anal. Chem..

[48] Giovannoli C., Baggiani C., Anfossi L., Giraudi G. (2008). Aptamers and molecularly imprinted polymers as artificial biomimetic receptors in affinity capillary electrophoresis and electrochromatography. Electrophoresis.

[49] Zhao Q., Wu M., Le X.C., Li X.F. (2012). Applications of aptamer affinity chromatography. Trends Anal. Chem..

[50] Cruz-Aguado J.A., Penner G. (2008). Determination of Ochratoxin A with a DNA Aptamer. J. Agric. Food Chem..

[51] Chapuis-Hugon F., du Boisbaudry A., Madru B., Pichon V. (2011). New extraction sorbent based on aptamers for the determination of Ochratoxin A in red wine. Anal. Bioanal. Chem..

[52] Ali W.H., Pichon V. (2014). Characterization of oligosorbents and application to the purification of Ochratoxin A from wheat extracts. Anal. Bioanal. Chem..

[53] De Girolamo A., McKeague M., Miller J.D., DeRosa M.C., Visconti A. (2011). Determination of Ochratoxin A in wheat after clean-up through a DNA aptamer-based solid phase extraction column. Food Chem..

[54] Rhouati A., Paniel N., Meraihi Z., Marty J.L. (2011). Development of an oligosorbent for detection of Ochratoxin A. Food Control..

[55] Yang X., Kong W., Hu Y., Yang M., Huang L., Zhao M., Ouyang Z. (2014). Aptamer-affinity column clean-up coupled with ultra high performance liquid chromatography and fluorescence detection for the rapid determination of Ochratoxin A in ginger powder. J. Sep. Sci..

[56] Wu X., Hu J., Zhu B., Lu L., Huang X., Pang D. (2011). Aptamer-targeted magnetic nanospheres as a solid-phase extraction sorbent for determination of Ochratoxin A in food samples. J. Chromatogr. A.

[57] Yang X., Hu Y., Kong W., Chu X., Yang M., Zhao M., Ouyang Z. (2014). Ultra-fast liquid chromatography with tandem mass spectrometry determination of Ochratoxin A in traditional Chinese medicines based on vortex-assisted solid-liquid microextraction and aptamer-affinity column clean-up. J. Sep. Sci..

[58] Brothier F., Pichon V. (2014). Miniaturized DNA aptamer-based monolithic sorbent for selective extraction of a target analyte coupled on-line to nanoLC. Anal. Bioanal. Chem..

[59] Merrifield R.B. (1963). Solid phase peptide synthesis. I. The synthesis of a tetrapeptide. J. Am. Chem. Soc..

[60] Gray B.P., Brown K.C. (2014). Combinatorial peptide libraries: Mining for cell-binding peptides. Chem. Rev..

[61] Righetti P.G., Candiano G., Citterio A., Boschetti E. (2015). Combinatorial peptide ligand libraries as a “trojan horse” in deep discovery proteomics. Anal. Chem..

[62] Li T.Y. (2006). Peptide and peptidomimetic chiral selectors in liquid chromatography. J. Sep. Sci..

[63] Tozzi C., Anfossi L., Giraudi G., Giovannoli C., Baggiani C., Vanni A. (2002). Chromatographic characterisation of an estrogen-binding affinity column containing tetrapeptides selected by a combinatorial-binding approach. J. Chromatogr. A.

[64] Tozzi C., Anfossi L., Baggiani C., Giovannoli C., Giraudi G. (2003). A combinatorial approach to obtain affinity media with binding properties towards the aflatoxins. Anal. Bioanal. Chem..

[65] Giraudi G., Anfossi L., Baggiani C., Giovannoli C., Tozzi C. (2007). Solid-phase extraction of Ochratoxin A from wine based on a binding hexapeptide prepared by combinatorial synthesis. J. Chromatogr. A.

[66] Tozzi C., Anfossi L., Baggiani C., Giovannoli C., Giraudi G. (2008). Synthetic peptides as artificial receptors towards proteins from genetically modified organisms. Biosens. Bioelectron..

